# Physical activity referrals in Swedish primary health care – prescriber and patient characteristics, reasons for prescriptions, and prescribed activities

**DOI:** 10.1186/1472-6963-8-201

**Published:** 2008-10-01

**Authors:** ME Leijon, P Bendtsen, P Nilsen, K Ekberg, A Ståhle

**Affiliations:** 1 Department of Medical and Health Sciences, Social Medicine and Public Health Science, Linköping Universitet, S-581 83 Linköping, Sweden; 2 Centre for Public Health Sciences, County Council of Östergötland, Linköping, Sweden; 3 Department of Medical and Health Sciences, National Centre for Work and Rehabilitation, Linköping Universitet S-581 83 Linköping, Sweden; 4 Department of Neurobiology, Health Care Sciences and Society, Division of Physiotherapy, Karolinska Institutet, Stockholm, Sweden; 5 Department of Medicine, Division of Cardiology, Karolinska Institutet, and Department of Physiotherapy, Karolinska Hospital, Stockholm, Sweden

## Abstract

**Background:**

Over the past decade, practitioners in primary health care (PHC) settings in many countries have issued written prescriptions to patients to promote increased physical activity or exercise. The aim of this study is to describe and analyse a comprehensive physical activity referral (PAR) scheme implemented in a routine PHC setting in Östergötland County. The study examines characteristics of the PARs recipients and referral practitioners, identifies reasons why practitioners opted to use PARs with their clients, and discusses prescribed activities and prescriptions in relation to PHC registries.

**Methods:**

Prospective prescription data were obtained for 90% of the primary health care centres in Östergötland County, Sweden, in 2004 and 2005. The study population consisted of patients who were issued PARs after they were deemed likely to benefit from increased physical activity, as assessed by PHC staff.

**Results:**

During the two-year period, a total of 6,300 patients received PARs. Two-thirds of the patients were female and half of the patients were 45–64 years. Half of the patients (50.8%) who received PARs were recommended a home-based activity, such as walking. One third (33%) of the patients issued PARs were totally inactive, reporting no days of physical activity that lasted for 30 minutes, and 29% stated that they reached this level 1–2 days per week.

The number of PARs prescribed per year in relation to the number of unique individuals that visited primary health care during one year was 1.4% in 2004 and 1.2% in 2005. Two-thirds of the combined prescriptions were issued by physicians (38%) and nurses (31%). Physiotherapists and behavioural scientists issued the highest relative number of prescriptions. The most common reasons for issuing PARs were musculoskeletal disorders (39.1%) and overweight (35.4%), followed by high blood pressure (23.3%) and diabetes (23.2%).

**Conclusion:**

Östergötland County's PAR scheme reached a relatively high proportion of physically inactive people visiting local PHC centres for other health reasons. PAR-related statistics, including PAR-rates by individual PHC centres and PAR- rates per health professional category, show differences in prescribing activities, both by patient categories, and by prescribing professionals.

## Background

Physical activity or the lack thereof is a major public health issue in many countries. The World Health Organization (WHO) has reported that physical inactivity is one of the 10 leading causes of death in developed countries, resulting in about 1.9 million preventable deaths worldwide annually[1]. Considerable knowledge has been accumulated over the past decades concerning the importance of physical activity in the treatment and prevention of a number of diseases [2-6]. Current guidelines to promote and maintain health recommend at least 30 minutes of moderate activity five days or more per week for adults between 18 and 65 years [7]. These guidelines have caused a shift from an exercise-fitness paradigm to a physical activity-health paradigm[8].

WHO states that promotion of physical activity is an important public health objective that requires a population-based approach, involving multiple sectors and disciplines [1,9]. To improve overall health in a community it is important to target the most inactive groups, as promotion of small increases in the activity levels among sedentary populations has a greater potential to influence public health than efforts aimed at increasing activity levels in those who are already active [10,11]. In many countries, primary health care (PHC) practitioners have implemented community-based schemes to improve activity levels, often referred to as exercise prescriptions or physical activity referral schemes [12-15]. Research demonstrates that physical activity referrals (PARs) prescribed by health care professionals in PHC settings can be effective in increasing patients' physical activity under controlled conditions [15-19]. Furthermore, physical activity and/or exercise prescriptions have been found to be acceptable and feasible both to general practitioners and the patients who receive exercise or physical activity recommendations via prescription [15].

In Sweden, the National Public Health Committee has identified PHC settings as key components in a multi-faceted community-oriented approach to promoting healthier lifestyles [20], as approximately 70% of the Swedish population consults health care providers at PHC centres each year [21]. Many Swedish PHC centres strive to go beyond basic health care to provide patients with education, counselling, and support programmes to bring about long-term improvements to health, such as increased physical activity, better nutrition, or smoking cessation [20]. While the use of PARs to increase physical activity is growing in many countries, including Sweden, little is known yet about the most effective way for practitioners to incorporate the use of such prescriptions in routine clinical practice [22]. There is also a paucity of research describing the characteristics of, and reasons for participation in PARs in the recipients of the prescription [13,23,24].

The aim of this study is to describe and analyse a comprehensive physical activity referral (PAR) scheme implemented in a routine PHC setting in Östergötland County during 2004 and 2005. The study examines characteristics of the PARs recipients and referral practitioners, identifies reasons why practitioners opted to use PARs with their clients, and discusses prescribed activities and prescriptions in relation to PHC registries.

## Methods

### Study setting

The study was conducted during 2004 and 2005 in Östergötland County, Sweden. This county of 416 000 inhabitants is the fourth largest region in Sweden and includes two larger cities (> 120,000 inhabitants) and 11 smaller, more rural municipalities. The county council maintains three hospitals and 42 PHC centres. All PHC centres in Östergötland County have a specified catchment area and/or a subscribed population. PHC staffs usually include different health care professionals, i.e. physicians, nurses, physiotherapists, occupational therapist, dieticians, and behavioural scientists (for example psychologists and mental health counsellors). The number of staff in the PHC centres in Östergötland County ranged from 10 to 80, with the number of physicians ranging from 2 to 12 and nurses from 8 to 35 (as of January 2005). At the end of 2003, 80% of the PHC centres in the region worked with PAR schemes to some extent, establishing a cooperative community-based structure to assist patients to gain access to various local activities.

The study analyses information collected in 37 (in 2004) and 38 (in 2005) of the 42 PHC centres operating within the county. Of the five centres that did not participate in 2004, two public PHC centres did not work with PARs and three private PHC centres declined to participate due to lack of time. In 2005, one of the two public non-participating PHC centres initiated a PARs scheme, and was included in this study.

Registry data show that all PHC centres in Östergötland County were visited by 234 250 unique individuals in 2004 and by 239 847 unique individuals in 2005. More females (55%) than males visited the PHC centres during the study period. With regard to age, 19% of the PHC visitors were 0–17 years, 12% were 18–29 years, 17% in the age group 30–44, 26% in the age group 45–64, and 26% were 65 years or older. More than half (56%) of the patients were seen by physicians, while 34% were seen by nurses during 2004 and 2005.

### Physical activity referral schemes in Östergötland County

The PAR schemes were first broadly introduced in Sweden in 2001 by the National Institute of Public Health in a national campaign called "Sweden on the move" [25]. The foundation of the Swedish PARs work was initially local and regional initiatives based on local networks and PHC professionals' own interests. Primary health care established collaborations with eligible physical activity organizations (i.e. local public health and sports organizations). The PARs coordinators/contact persons were appointed both at the PHC centres and participating physical activity organizations[25].

In Östergötland County, information packages were assembled for use by the members of the regional PARs network containing patient materials describing the health benefits of physical activity, waiting room posters for PHC centres, and referral forms for participating PHC personnel. A previously existing economic incentive to promote improved health care quality in PHC (e.g. telephone lines for non-urgent health advice, systematic asthma care) was targeted to PARs in the county in 2004. Experiences with this patient-oriented approach resulted in the county council's introduction of incentives to support PARs work in general, to stimulate prescription activity, and to compensate for the extra amount of work required to collect and assemble prescription data during the study period. The incentives, which primarily involved additional operating funds to participating PHC centres, required the PHC centres to issue a pre-determined minimum number of prescriptions, ranging from 50 to 100, depending on the size of the PHC centres. The incentives also required that participating PHC centres designate coordinators responsible for collecting baseline and follow-up statistics about the PAR scheme in each PHC centre.

Patients eligible to receive PARs in participating PHC centres were those whom staff believed would benefit from increased physical activity, due to sedentary lifestyles and-or diagnoses indicating that increased physical activity could be beneficial, e.g. high blood pressure, diabetes, and musculoskeletal disorders. Participating patients were provided with written prescriptions and copies were kept in patients' medical records. If an activity prescribed was facility-based, a copy of the PAR was also sent to the PARs coordinator in the selected physical activity organization, who then contacted the patient by telephone or letter. Referred patients paid normal participation fees to the organizations for the activities they attended. In addition to structured facility-based activities, PARs also included recommendations for self-initiated, home-based activities such as implementing a regular schedule for walking outdoors.

### Data collection

Information about patient visits, recorded in PHC registries and PARs related information were collected by the PAR coordinator in each PHC centre. All PARs prescription forms were registered in a Microsoft Excel-based spreadsheet, which was sent to the first author three times a year for ongoing analysis.

Prescription forms designed for the data collection covered background information about patients (age, sex, address, and telephone numbers) and their current activity levels: number of days with at least a total of 30 minutes of physical activity the previous week (7-day recall) and a normal week. Reasons for prescribing PARs were registered in the prescription forms by health care providers checking one or more of seven pre-defined categories including sedentary lifestyle, or pre-existing diseases including known risk factors related to lack of physical activity: musculoskeletal disorders; overweight (defined as a body mass index > 25); diabetes; high blood pressure; high blood cholesterol; and mental ill-health. The category also included a free-text line to justify the PAR. Free-text responses were categorised and re-coded into new categories. However, the numbers in each category were small and are presented in tables as "other reasons".

### Analysis

The study population was divided into age groups 0–17, 18–29, 30–44, 45–64 and 65 year and older, age groups that are normally used in reports and registry data in Östergötland County. A PARs-rate was calculated from the number of PARs issued in PHC during one year divided by the number of unique individuals who visited the 42 PHC centres in the region each year. A practitioner specific -PAR rate was calculated from the number of PARs issued by different types of health care providers during one year and presented as proportions: the total number of PARs divided by the number of unique individuals that visited each health care provider category during one year.

Health care providers' reasons for PARs are presented in this study as proportions of all patients receiving PARs. As many patients had multiple reasons for their prescription, the total proportion of reasons for prescriptions published in this study exceeds 100%, as does prescribed activity types. The statistical software SPSS (release 14.0) was used for all analyses.

## Results

The average number of PARs per PHC centres was 90 prescriptions in 2004 (with a range of 42–182 prescriptions) and 78 prescriptions per PHC centres in 2005 (with a range of 20–154 prescriptions). There were substantial seasonal variations in PARs, with the highest number of prescriptions issued from February to April and the lowest numbers during July.

### Patient characteristics

Table 1 describes sex and age distribution of PARs patients in participating PHC centres in Östergötland County in 2004 and 2005, and presents PAR rates during the study period- i.e. the number of prescriptions issued per year in relation to the number of unique individuals who visited PHC during one year. During the two-year period, a total of 6,300 patients received PARs (3,344 in 2004 and 2,956 in 2005), amounting to about 1.5% of the total county population. The average age of PARs patients was about 54 years. The youngest patient was 12 years old and the oldest was 96 years old. Females had higher total PAR rates than males, with an average of 1.6% compared to 1.0% for men during this two-year period. Patients between 45 and 64 years of age had the highest PAR rate, with an average of 2.5% of the visits (by unique individuals) in 2004 and 2005.

**Table 1:** Physical activity referral rates in relation to sex and age

	2004		2005		Total	
	Number (%)	PARs-rate	Number (%)	PARs-rate	Number (%)	PARs-rate

**Sex**						
Female	2218 (66.3)	1.7	1972 (66.7)	1.5	4190 (66.5)	1.6
Male	1125 (33.6)	1.1	983 (33.3)	0.9	2108 (33.5)	1.0
**Age group**						
0–17	13 (0.4)	0.03	19 (0.6)	0.04	32 (0.5)	0.04
18–29	160 (4.8)	0.5	183 (6.2)	0.6	343 (5.4)	0.6
30–44	716 (21.4)	1.8	556 (18.8)	1.4	1272 (20.2)	1.6
45–64	1683 (50.3)	2.7	1449 (49.1)	2.3	3132 (49.7)	2.5
65+	771 (23.1)	1.3	747 (25.3)	1.2	1518 (24.1)	1.2

**Total**	3344	1.4	2956	1.2	6300	1.3

Note: The PARs-rates are the number of PARs issued in PHC during one year divided by the number of unique individuals that visited PHC in one year.

Patient records showed strong variations in activity levels before PARs were given by practitioners. When asked to recall physical activity in the immediate past seven days and over a 'normal' week, the proportion of inactive recipients, i.e. those who reported no activity, was 33% and 27% respectively. Another large proportion, (29% for a 7-day recall and 30% for a 'normal' week) reported only 1–2 days a week where their physical activity lasted at least 30 minutes. Only a fourth of patients stated that they were already regularly active, reporting 5–7 days where physical activity lasted for at least 30 minutes, (22% (7-day recall) and 24% (normal week) respectively).

### Primary health care practitioners

The specific health professions of practitioners who issued PARs in 2004 and 2005 are shown in table 2. Overall, nearly two-thirds of the prescriptions were issued by physicians (35% of all PARs issued) and nurses (30%). However, the profession-specific PAR rate shows that physiotherapists and behavioural scientists produced the highest relative number of prescriptions, i.e. the number of prescriptions issued in relation to the number of unique individuals that visited each professional category. The results for 2004 and 2005 were similar, although physiotherapists displayed somewhat higher rates and physicians slightly lower rates in 2005 compared to 2004.

**Table 2:** Physical activity referral rates by referring health practitioners

	Prescriptions n (%)		Professional-PARs rate (%)	
	2004	2005	2004	2005

Physician	1238 (38.1)	904 (31.5)	0.6	0.5
Nurse	1022 (31.4)	814 (28.4)	0.9	0.7
Physiotherapist	504 (15.5)	610 (21.3)	14.6	22.1
Occupational therapist	53 (1.6)	31 (1.1)	0.6	0.7
Dietician	129 (4.0)	168 (5.9)	3.9	4.6
Behavioural scientist	62 (1.9)	36 (1.3)	10.5	15.6
Other	245 (7.5)	303 (10.5)	1.3	1.3

Total	3253 (100)	2866 (100)	-	-

Note: The profession- PAR- rate is a ratio expressing the number of PARs issued by different professional categories in one year divided by the number of unique individuals that visit each category group each year.

There was considerable variation in the proportion of PARs issued by specific professional categories when viewed by individual PHC centres. In some centres, only 4% of the prescriptions were issued by physicians, while in one particular centre, physicians issued all of the prescriptions. The proportion of PARs issued by nurses ranged from 0% to 93%; the proportion of PARs issued by physiotherapists ranged from 0% to 66%.

### Reasons for physical activity referral

The most common reasons for issuing PARs included musculoskeletal disorders (39.1%) and overweight (35.4%), followed by high blood pressure (23.3%) and diabetes (23.2%) (see Table 3). Females who received PARs had higher proportions of referrals related to musculoskeletal disorders than males. Prescriptions for males were more likely to cite diabetes and high blood pressure as motivators for PARs.

**Table 3:** Reasons for physical activity referral

	Sex		Age group					Profession		Total
	Female (n = 4190)	Male (n = 2108)	0–17 (n = 32)	18–29 (n = 343)	30–44 (n = 1272)	45–64 (n = 3132)	65+ (n = 1518)	Physician (n = 2142)	Other (n = 3977)	n = 6300
	(%)	(%)	(%)	(%)	(%)	(%)	(%)	(%)	(%)	(%)

Reasons for prescriptions*										
Sedentary	14.6	13.9	21.9	22.7	17.1	13.0	12.8	17.7	12.9	14.4
Musculoskeletal	44.6	28.8	43.8	40.5	46.0	37.8	35.7	37.2	40.4	39.1
Overweight	35.2	35.7	37.5	42.9	38.5	36.2	29.2	39.4	33.8	35.4
Diabetes	18.3	32.9	6.3	1.7	7.5	26.2	35.2	17.0	27.2	23.2
High blood pressure	20.4	28.9	0	1.7	10.1	27.9	30.2	25.3	22.7	23.3
Cholesterol	8.0	10.2	0	0.3	2.5	10.6	12.2	9.8	8.5	8.7
Mental health	10.1	7.4	3.1	23.0	16.2	8.1	2.4	14.1	6.9	9.2
Other reasons	9.4	8.0	15.6	9.0	7.4	8.4	11.1	10.1	8.3	8.9

Notes:

*) The total sums exceed 100%.

Prescriptions due to high blood pressure, high blood cholesterol and diabetes were positively associated with older age. The number of patients issued prescriptions due to multiple health reasons increased with patients' ages. Free text describing other reasons for prescribing physical fitness activities consisted primarily of asthma and chronic pulmonary disease (n = 63).

'Mental ill-health' as a reason for PAR was most common among those aged 18–44. The number of patients who were issued PARs due to mental ill-health grew during the study period from 7.8% (n = 262) in 2004 to 10.7% (n = 317) in 2005. Mental ill-health was cited as a justification for PARs more often for females as the study period progressed, increasing from 8.5% (n = 188) in 2004 to 12% (n = 236) in 2005.

'Being sedentary' as a primary reason or in combination with other reasons for PARs, was a prescribing justification used most commonly for patients in younger age groups. 'Being sedentary' was more frequently issued as a justification for PARs by physicians than all the other professional groups combined. Physicians also more frequently issued PARs to patients with overweight and mental health problems. The other practitioner categories to a larger extent justified PARs to patients due to musculoskeletal disorders and diabetes.

### Prescribed type of physical activity

Table 4 describes the various types of activities that were prescribed to the recipients. Half of the patients (50.8%) were prescribed a home-based activity such as walking, which was the most common activity prescription for both sexes and for all age groups. Structured group-based activities, including water aerobics, group gymnastics and Nordic walking in groups, were more commonly prescribed to females than to males. Gymnastics and weight and circuit training were more commonly prescribed to younger patients,

**Table 4:** Prescribed type of physical activity for referred patients

	Sex		Age group					Profession		Total
	Female (n = 4190)	Male (n = 2108)	0–17 (n = 32)	18–29 (n = 343)	30–44 (n = 1272)	45–64 (n = 3132)	65+ (n = 1518)	Physician (n = 2142)	Other (n = 3977)	n = 6300
	(%)	(%)	(%)	(%)	(%)	(%)	(%)	(%)	(%)	(%)

Prescribed activity*										
Walking	46.7	58.9	37.5	37.9	42.8	52.6	57	51.5	50.8	50.8
Nordic walking**	11.6	6.2	0	4.1	7.2	10.8	11.3	12.7	8.3	9.8
Running	0.7	1.5	6.3	1.7	1.8	0.8	0.2	1.7	0.6	1.0
Swimming	3.6	5.1	12.5	7.3	5.4	4.1	2.2	5.2	3.6	4.1
Water aerobic	31.0	12.9	6.3	19.2	22.7	25.5	27.1	21.2	26.5	24.9
Group Gymnastics	14.8	9.6	9.4	20.4	17.2	11.9	10.4	17.9	12.5	13.1
Weight & Circuit	17.3	21.4	40.6	33.5	26.7	17.6	10.6	17.6	19.8	18.7
Other activity	26.4	29.6	31.3	29.7	30.1	27.4	24.8	27.8	25.8	27.5

Notes:

*) The total sums exceed 100%.

**) Nordic walking, also known as ski walking, pole walking or fitness walking, involves walking with modified ski poles.

## Discussion

This study describes and analyses characteristics of a PAR scheme implemented in a county-based health care system in Sweden. The study shows an annual PAR rate of 1.4% in 2004 and 1.2% in 2005, meaning that one in every 70 to 80 PHC patients visiting PHC in the study area was prescribed physical activity. Similar levels of prescription rates have been reported in previous studies of PARs or exercise referral programmes in other geographically defined populations [13,14,26]. There is a paucity of population-based studies and research describing PARs characteristics, including the whole range of physical activity advice given in PHC settings [23,24]. Certainly, many practitioners will provide information about the need for physical activity during patient visits, without issuing PARs, making it even harder to evaluate these kinds of concepts. The seemingly low prescription rates evidenced in this study may also be explained by the fact that PARs was a relatively new programme during the study period. On the other hand, it is not possible to determine how many patients would have received PARs or advice about physical activity in the absence of the introduction of the PARs programme.

Most of the available research concerning PARs is limited to written prescriptions issued primarily by physicians [24]. The study described here highlights the use of PARs by different health care practitioners in clinical settings. The importance of involving allied health professionals in PHC-based PAR schemes has been demonstrated in previous research [26-28]. Different approaches by the various health care practitioners almost certainly influenced both the number of prescriptions and the distribution of reasons for referring patients to physical activities in Östergötland County. Physicians and nurses issued the majority of the prescriptions in this study. However, physiotherapists and behavioural scientists issued the highest number of PARs in relation to the number of unique individuals that visited each professional category. Still, in this study, it was physicians who met the majority of patients who visited PHC centres. Physicians issued the greatest number of referrals, and also the highest proportion of PARs to inactive patients, which underscores the critical role of physicians in the PARs approach to achieve increased levels of physical activity in the community.

There were large variations in the proportions of prescriptions issued by different types of PHC practitioners at different PHC centres, showing that the PAR scheme still is under development in Östergötland County. In some PHC centres, all staff members were given the opportunity to issue referrals, whereas in one centre, only physicians issued referrals. The PAR scheme was designed to be flexible, allowing PHC practitioners and centres to tailor the work according to their local conditions. However, given the wide range in PARs issued by different practitioner categories among the participating PHC centres, there is clearly an opportunity for increased activity by some practitioners in many centres. The general PARs rate and profession-specific PARs rates were constructed during this study to allow for monitoring of differences in prescription levels. The measures can also be used by individual PHC centres in order to identify areas for improvement and modify goal setting.

The study results demonstrate that the intervention primarily reached physically inactive individuals. Almost one third of the recipients (about 2000 patients) who received a referral for physical activity were categorised as sedentary. By comparison, national data indicate that approximately 14% of both sexes aged 18–84 in Sweden as a whole were categorised as physically inactive or sedentary in 2004 [29]. The referrals appeared to provide a good method for health care practitioners to stress the importance of physical activity amongst community members least likely to engage in such activities.

While many of the prescriptions in this study were based on poor physical health, we note the relatively high numbers of referrals issued due to poor mental health, particularly to younger adults in the 18–44 age category. Heightened media interest in mental ill-health in Sweden and an increased awareness of the association between mental health and physical activity may partially explain the high numbers of PARs for mental health reasons. The justifications for assigning patients to exercise due to poor mental health were vague in this study- the category was simply defined as such, mental ill-health. With such a broad definition, it is likely that many prescribing health professionals could justify including a patient in this category

We note also that many patients who already stated they were active received PARs. In these cases, it may have been that a change from one activity to another was advised, due to muscle strains or other physical problems. It is also possible that activity changes were suggested in order to increase the amount of exertion expended by patients.

To a great degree, the reasons for justifying PARs and resultant prescribed activities were associated with the age distribution of patients. Accordingly, patients with high blood pressure, high blood cholesterol, and diabetes were positively associated with older patient ages and there was a positive relationship between age and patients who were categorized as having multiple reasons justifying PARs. Younger patients, ages 44 and under, were usually prescribed more physically strenuous activities such as group gymnastics and weight and circuit training, while activities such as walking, Nordic walking, and water aerobics were more common among the older age categories. Females to a much larger extent than males were prescribed group-based activities such as water aerobics and group gymnastics. Walking was the most frequently prescribed activity for both sexes and in all age categories. This finding is consistent with recent physical activity guidelines, which has resulted in walking becoming something of a gold standard for low-intensity physical activity [6,30]. Numerous studies have demonstrated that walking is a feasible activity for sedentary individuals and a valuable activity for health enhancement [2-5,31].

This study has limitations in generalisability. We have limited information about some important factors affecting patients, e.g., why they visited their PHC centres, their overall health status or socioeconomic status. In addition, there are environmental factors that probably affect the study's outcomes. The economic incentives in the study regions to support PARs may be considered an intervention in itself, as these incentives could have enhanced the PAR effort and yielded a higher number of prescriptions than would otherwise have been the case. Although studies involving economic incentives have so far showed mix results on the provision of preventative services [32,33], it is possible that the incentive schemes did impact practitioner willingness to prescribe physical fitness activities. As part of the county's incentive scheme, goals were set for the total number of prescriptions to be issued, which may have resulted in PHC centres only trying to attain these stated goals. Another weakness of the findings is that PAR rates were derived from the number of prescriptions from the included PHC centres in relation to all PHC centres in the region, which could have led to underestimates of the rates at the PHC centre level. However, the number of non-participating clinics was small (10 percent), and should not impact rates to a significant degree. This study took place in a particular setting where universal access to health care is a right; as access to health care systems differ between countries, results are not necessarily applicable and easy to translate between countries and health care systems [34]

A considerable strength of the study was its real-life implementation. All prescriptions were made by ordinary staff in their normal workplaces. A high proportion of PHC centres (90%) in the study location participated, which makes it possible to generalise findings to other PHC settings, at least in a Scandinavian context. Only small differences in the number of prescriptions were seen over the two study years, indicating that data were reliable.

While lifestyle interventions tend to focus on the individual responsibility for health behaviours, the PAR scheme in Östergötland County was designed as a community-based effort. The guiding principle of the programme was collaboration between the PHC centres and local physical activity organisations in the region. The health care system, and its practitioners, has an important role in developing local and regional PARs due to its professional knowledge, authority, and reach in the population. If community-level health improvement is a goal, it is important to apply a social ecological perspective that views physical activity as a result of a multitude of influences at different levels [35]. People's health behaviours reflect their life experiences and these experiences are determined by broader institutional structures, cultural forces, and social relations within the community [36]. In the long run, it would be desirable to expand on the present concept by integrating PAR efforts with interventions targeting multiple risk and/or behaviours, including tobacco use, alcohol, and healthy eating, as most people who suffer from life-style related health issues present with more than one risk factor and/or unhealthy behaviour [37]. Moreover, a broader, more all-encompassing prescription-based programme might be more feasible to integrate in everyday PHC practice, as lifestyle interventions then would be viewed as a single concept rather than isolated efforts competing for practitioner's attention and resources.

## Conclusion

The PAR scheme in Östergötland County reached one in 70 to 80 PHC patients visiting PHC in the study area and a relatively high proportion of physically inactive people. Females and middle aged patients were to a larger extent issued PARs. The Physical Activity Referral scheme appeared to target the appropriate groups of patients, where physical activity could indeed promote improvements to health. Physicians and nurses issued PARs to the highest numbers of patients, but in relative figures the physiotherapists and behavioural scientists issued the highest number of patients. Primary health care based physical activity intervention is still relatively new in Sweden and there are many avenues for growth and improvement in the years to come.

## Abbreviations

PAR: Physical activity referral; PHC: Primary health care

## Competing interests

The authors declare that they have no competing interests.

## Authors' contributions

MEL the first author led the study design, oversaw data collection, analysed data on a regular basis, worked with the study statistician to interpret findings, and organized the drafts of this article. PB was materially involved in development of this article's first, second, and final draft and interpretation of data. Has final approval rights to the submitted version of the article. PN has been materially involved in the second and final drafts of this article, and has made critical and important contribution to the organisation of the manuscript, and has final approval rights to the submitted version of the article. KE have been involved in the second and final drafts, with special attention to the methods section. Author has final approval rights to the submitted version of the article. AS was materially involved in development of this article's first, second, and final draft and interpretation of data. Has final approval rights to the submitted version of the article.

## Funding

County Council of Östergötland

## Ethical approval

Not required – data collection was part of the health care routine system

## Pre-publication history

The pre-publication history for this paper can be accessed here:


